# The Impact of the COVID-19 Pandemic on the Development of Motor Skills of German 5- to 6-Year-Old Children

**DOI:** 10.3390/bs15030353

**Published:** 2025-03-13

**Authors:** Aileen Kotzsch, Andy Papke, Angela Heine

**Affiliations:** 1 SportService des Landessportbund Brandenburg e.V., 14471 Potsdam, Germany; 2 Department of Psychology, University of Duisburg-Essen, 45141 Essen, Germany

**Keywords:** motor skills, development, preschool children, COVID-19 pandemic

## Abstract

The closure of educational institutions, playgrounds, and sports facilities has had a significant impact on children’s levels of physical activity during the COVID-19 pandemic. Currently, there are only a few studies available that address the consequences of these pandemic-related restrictions on the development of motor skills in younger children. The aim of the present study was to gain insights into the impact of the COVID-19 pandemic on the development of children’s fine and gross motor skills by comparing cohorts of German preschoolers. For this purpose, data from annual (years 2015 to 2018 and 2020 to 2024) routine examinations of children’s (*n* = 1426; mean age: 5.46 ± 0.29 years) motor development conducted in the child daycare centers of the SportService des Landessportbund Brandenburg e.V. [Federal Sports Association of Brandenburg, Germany] were subjected to a secondary analysis. While no effects on overall motor performance as measured by the MOT 4–6, a standardized motor skills test, were found for the groups of preschoolers, a more detailed analysis revealed that the cohorts of children differed with respect to certain motor domains, i.e., jumping performance and fine motor skills. The findings are relevant for designing targeted remedial measures for children affected by the pandemic-related restrictions.

## 1. Introduction

One focus of research in human movement science is the development of fundamental motor skills (Jones et al., 2020; Logan et al., 2015; Schott & Voelcker-Rehage, 2023), which serve as the building blocks for more complex and context-specific motor behavior. Stodden et al. (2008) suggest regular physical activity, both in the context of free play and targeted exercise programs, to be essential for the development of fundamental motor skills in childhood. So, as the earliest institutionalized educational contexts, child daycare centers play a crucial role in promoting an active childhood and, thus, in the development of basic motor skills.

In line with this, the WHO recommendations published in 2020 (Bull et al., 2020) emphasize the importance of daily physical activity during childhood. For children around the age of 5, 180 min of physical activity are recommended daily, with at least 60 min of moderate to vigorous intensity. Despite these recommendations, however, physical activity among children has declined significantly in recent years. According to the KiGGS study (Finger et al., 2018), less than one-third of children and adolescents in Germany meet the WHO recommendations. International studies show similar trends (for an overview, see Aubert et al., 2022). This general decline in physical activity has significant implications for the motor development of young people, particularly in the early years when the basis for fundamental motor skills is laid. As expected, various studies show a connection between decreasing physical activity and reduced motor skills in children (Jaakkola et al., 2016; Opper et al., 2007; Tiemann, 2021). This general trend has been further exacerbated by the COVID-19 pandemic. Not surprisingly, the impact of the closures of daycare centers, schools, playgrounds, and parks on both children’s physical activity and, as a consequence, their motor development has been the subject of a number of studies.

### 1.1. Impact of the COVID-19 Pandemic on Children’s Physical Activity Levels

A Canadian study found that during the pandemic, 87.5% of children and adolescents aged 5 to 17 years did not achieve the recommended 180 min of physical activity per day (Moore et al., 2020). Similar results were found in a study by Dunton et al. (2020) in the United States, which also identified a significant decline in physical activity among children aged 5 to 13 years, with children aged 9 to 13 years showing a greater decline in physical activity than younger children (5 to 8 years). In an Italian study, Pietrobelli et al. (2020) reported that children aged 6 to 18 years spent more time sitting and participated less in sports activities during the pandemic. For Chinese children aged 6 to 17 years, a study by Xiang et al. (2020) found that the time spent outdoors decreased significantly during the COVID-19 pandemic, resulting in an overall decline in daily physical activity. In addition, a study by Haile et al. (2023) showed that Swiss children and adolescents were also less physically active. In the early phases of the pandemic, only 59% of the primary school children and 52% of the secondary school children met the WHO recommendations (Bull et al., 2020) for physical activity. So, due to the closure of educational institutions, playgrounds, and sports facilities, the COVID-19 pandemic has led to a global period of physical inactivity of unprecedented proportions, the long-term effects of which on children’s physical fitness and motor performance have not yet been sufficiently investigated (Graber et al., 2021).

Although a general decline in physical activity during the COVID-19 pandemic was reported by all studies, a look at the literature on children’s motor performance development reveals a more nuanced picture of the pandemic’s impact. While some studies found no or only minor changes in motor performance levels, others reported significant differences in various motor parameters—sometimes to different degrees depending on age group, socioeconomic background, and the motor dimensions examined. To provide an overview of the current state of research, studies with school children will be considered first, followed by studies that examined children of preschool age.

### 1.2. Impact of the COVID-19 Pandemic on the Motor Performance Levels of School Children

In a longitudinal study conducted in the Netherlands with 992 primary school children aged 6 to 12 years, whose motor performance was documented over several years using a skills test that included four dimensions, Den Uil et al. (2023) found no significant long-term deterioration in children’s developmental trajectories. Although initially, differences in pandemic-affected in contrast to control cohorts were discernible, a return to pre-pandemic levels was demonstrated for later measurement points. Similar results were reported by Carballo-Fazanes et al. (2022) for Portuguese children (*n* = 67; 7.4 to 12.2 years) who were assessed in the years between 2018 and 2022 using a motor competence test. Despite the pandemic-related restrictions, no significant impact on children’s motor skills was found. Similarly, a Spanish study by López-Bueno et al. (2021), which examined the endurance performance of 12- to 14-year-old school children using the 20 m shuttle run test before and after the lockdowns, reported no statistically significant effects.

In contrast, there are several studies that did find the effects of the COVID-19 pandemic on the motor skills of school children. A large-scale German study by Stojan et al. (2023), which included motor test data from 68,996 third-graders from 2011 to 2023, showed inhomogeneous effects, i.e., while certain motor skills such as the 20 m sprint and push-ups deteriorated during the pandemic, endurance performance such as 6 min run performance improved. Furthermore, the authors reported the socioeconomic background of the children to have an influence. Children from higher socioeconomic strata were more likely to show negative changes, while children with a socially less advantaged background showed less of a decline or even improvement in motor performance. Comparing data from the years 2016, 2020, and 2021, another German study (Wessely et al., 2022) reported an increase in children’s body mass index and a decrease in motor test performance (e.g., 6 m sprint, sideways jumping), which was particularly pronounced in children from socially disadvantaged neighborhoods. A general decline in fitness parameters was shown by Chambonnière et al. (2021) for 206 French primary school children (9 to 10 years old) who were examined between February 2020 and January 2021. A deterioration of anthropometric and motor parameters, such as maximum aerobic speed, jumping power, and hand strength, was reported. For English school children, Basterfield et al. (2022) found improved standing jump and handgrip strength but a decline in shuttle run endurance during the pandemic in a group of 8- to 10-year-old children from disadvantaged areas. Similarly, Eberhardt et al. (2022) found short-term improvements in motor performance in German school children. An early COVID-19 cohort showed improved jumping performance compared to pre-COVID-19 levels. When comparing a pre-COVID-19 to a late COVID-19 cohort, the performance data converged. Studies from China (Xiang et al., 2020), Slovenia (Pajek, 2022), Austria (Greier et al., 2020), the Netherlands (Vrieswijk et al., 2021), and two further German studies (Joisten, 2022; Wunsch et al., 2021) also reported complex patterns of effects of the COVID-19 pandemic on various aspects of children’s motor performance. What is more, Pombo et al. (2021) found significant gender differences in pandemic-related effects on children’s motor performance. The study examined physical fitness and motor skills before and after the COVID-19 lockdown in Portugal. Initially, 182 children aged 7 to 11 were tested for their motor skills, with 114 of them being re-examined after the lockdown. The study not only showed that the restrictions on physical activity imposed by the lockdown had an overall negative impact on children’s motor development but also that boys, in particular, showed a significant deterioration in both the long jump from a standing position and running speed, while throwing performance was particularly affected in girls. In conclusion, on the one hand, significant losses were found across these studies, for example, with respect to endurance and speed parameters. On the other hand, increases or at least stability in certain parameters were observed, too. In general, younger children or those with less motor skills showed more pronounced impairments, while older children or those with more developed motor skills were found to be less affected by the pandemic-related restrictions. Socioeconomic factors were reported to also play a role.

### 1.3. Impact of the COVID-19 Pandemic on the Motor Performance Levels of Preschool Children

While there is a comparatively large amount of data available for school children, there are fewer studies on preschool children (3 to 5 years). For this age group, no consistent trend can be identified either. For example, Abe et al. (2022) examined running, jumping, and throwing performance in a Japanese cohort of 3- to 5-year-olds, with baseline data collection in 2019 and a retesting in 2020. They found that 5-year-olds performed worse in the 25 m sprint after the start of the pandemic, while no significant effects were found for younger age groups. Additionally, there were slight declines in children’s ability to control an object (softball throw) in all age groups. Performance in the standing long jump remained largely stable. For Germany, Rigó and Weyers (2024) used data from the regular school entry examinations. Although the results are not reported in detail, the authors pointed out coordinative deficits as well as a deterioration in long jump performance. Quenzer-Alfred (2024) examined German preschool children (3 to 6 years old) before, during, and after periods of strict lockdown in a cross-sectional study. Motor skills (e.g., running, jumping, balancing, and fine motor skills) were assessed using standardized tests. Parent questionnaires provided additional information on physical activity opportunities in the home environment. The results showed a significant decline in children’s fundamental motor skills during the lockdown phases, particularly for children with limited access to outdoor activities and children from socioeconomically disadvantaged families. Some improvements were observed after the end of the pandemic restrictions, but not all pandemic-related deficits were fully compensated.

Overall, these studies also point to rather complex patterns of the effects of the pandemic on children’s motor development. What makes identifying systematic trends difficult is that regional differences in lockdown measures may have contributed to the apparent heterogeneity of results. Furthermore, the available data suggest that some motor dimensions may be more sensitive to pandemic-related restrictions than others and that some groups of children may be more affected than others. In summary, the current state of the literature underscores the need for further studies to assess the impact of pandemic-related restrictions on children’s motor development.

Hence, the aim of the present study was to gain further insights into the effects of the COVID-19 pandemic on children’s fine and gross motor skills. For this purpose, existing anonymized data from regular assessments of children’s levels of motor development that were conducted at the daycare centers of the SportService, a subsidiary of the Landessportbund Brandenburg e.V. (Federal Sports Association of Brandenburg, Germany), were subjected to a secondary analysis. Cohorts of German preschoolers from the years 2015 to 2018 and 2020 to 2024 were compared in a cross-sectional study to add to the rather limited existing literature on the impact of pandemic-related restrictions in physical activity on younger children’s motor development.

### 1.4. Chronology of COVID-19 Pandemic-Related Restrictions in Germany

The COVID-19 pandemic has significantly shaped public life in Germany since the beginning of 2020, when the first case of infection in Germany was detected in Bavaria on 27 January (Tolksdorf et al., 2022). The pandemic situation in Germany escalated with the first official death on 8 March 2020 and when Heinsberg, a town in North Rhine-Westphalia, developed into one of the first corona hotspots in Europe. To contain the spread, the German federal government took extensive measures. On 22 March, a nationwide curfew went into effect, resulting in the closure of schools, daycare centers, and other public institutions (Tolksdorf et al., 2022). At this point in time, people began to avoid social contact, increasingly worked from home, and stayed away from public events. In the summer of 2020, decreasing infection rates led to a gradual relaxation of the restrictions. However, infection rates increased again in the fall of 2020. So, lockdown measures were tightened again in December 2020, leading to the second closure of daycare centers and schools (Schilling et al., 2021). At the end of December 2020, a gradual relaxation of the rules for participation in public life started. In Brandenburg, preschool facilities gradually reopened after the initial COVID-19 closures.

## 2. Materials and Methods

### 2.1. Participants

For the present study, existing anonymized data from children aged between 5.0 and 6.3 years from eight child daycare centers of the SportService des Landessportbund Brandenburg e.V. were used (*n* = 1426; cf. Table 1). As part of the standard diagnostic assessment program for all children enrolled in the daycare centers, development of gross and fine motor skills was screened around the time they left kindergarten for school. For this purpose, the MOT 4–6 (Motoriktest für vier- bis sechsjährige Kinder [Motor Proficiency Test for children 4 and 6 years old], Zimmer, 2015; Zimmer & Volkamer, 1987), a standardized diagnostic test battery, was used. Assessment data from children’s last kindergarten years from 2015 to 2018 and from 2020 to 2024 were available for secondary analysis. Data from 2019 are missing due to a temporary change in diagnostic assessment procedures at the daycare centers.

Upon enrolling their children in one of the daycare centers, parents and/or other primary caretakers give written informed consent with respect to all procedures related to data collection and analysis carried out in the context of the regular screening procedures implemented in the facilities of the SportService des Landessportbund Brandenburg e.V. Written parental consent for the secondary use of anonymized assessment data is given separately. All data collection procedures are conducted in accordance with the Declaration of Helsinki. The processes related to the collection, anonymization, handling, and analysis of the data were approved by an independent external data protection officer.

### 2.2. Procedures

Children were tested in the context of their respective daycare centers. Children’s gross and fine motor developments were assessed using the MOT 4–6 (Zimmer, 2015; Zimmer & Volkamer, 1987). Each child was individually tested by a person from a team of trained diagnosticians. The children performed all tasks of the MOT 4–6 under the guidance of the respective diagnostician, who adhered to the standardized instructions for the application of the MOT 4–6 (Zimmer, 2015; cf. test manual). The individual MOT 4–6 testing session lasted between 20 and 30 min.

### 2.3. Instruments

The MOT 4–6 is one of the most commonly used standardized German test batteries for assessing the motor development of typically developing preschool children (Wagner et al., 2011). In addition to its main fields of application within developmental, educational, and sports sciences, Nestler and Castello (2003) point out the importance of the MOT 4–6 as an instrument that is also used by practitioners in, for instance, German educational counseling centers.

The MOT 4–6, which was first published in 1987 (Zimmer & Volkamer, 1987), was revised and re-normed recently (Zimmer, 2015). Overall, good psychometric properties are reported for MOT 4–6 (stability: r = 0.85; internal consistency: α = 0.81; cf. Zimmer, 2015; Wagner et al., 2011). The diagnostic assessment is based on a quantitative and qualitative evaluation of children’s motor development with regard to 17 diagnostic tasks, which load on seven sub-scales (cf. Table 2).

Children’s motor performance in each of the tasks of the MOT 4–6 is assessed using a standardized rating scale on the basis of which the diagnostician assigns scores between 0 (complete failure) and 2 points (maximum performance). The MOT 4–6 provides a detailed scoring scheme (cf. Instruction Handbook of the MOT 4–6; Zimmer, 2015), which specifies not only the global motor behavior goal for each task but also relevant aspects of task execution that affect performance. In addition to the quantitative evaluation, qualitative assessment of the children’s performance and behavior can be carried out for each task using standardized observation protocols. To guarantee that the child understands the respective task correctly, before running through a certain task of the MOT 4–6, the diagnostician first explains and demonstrates correct performance and then practices task execution with the child.

### 2.4. Preprocessing of the Data and Data Analysis

All data were digitized. For each child, the latest possible data point was selected for analysis by the first author (A.K.). Children’s MOT 4–6 composite raw scores for each of the seven sub-scales divided by the number of subtests for the respective scale, as well as age-normed T-scores for mean overall motor performance, were analyzed statistically using SPSS Statistics for Macintosh, Version 28.0 (IBM Corp, 2021). Age was converted into a categorial variable with three levels (younger: 5.0 to 5.3 years; middle: 5.4 to 5.7 years; older: 5.8 years and older). The data were checked for outliers, and normality was visually confirmed for the subgroups using P-P plots.

First, a three-way ANOVA was performed to examine the effects of cohort (factor levels: pre-COVID, COVID 1, COVID 2, post-COVID 1, post-COVID 2; cf., e.g., Eberhardt et al., 2022), gender (factor levels: male, female), and age (factor levels: young, middle, old) on overall MOT 4–6 T-scores. Homogeneity of variances was determined using Levene’s test [*F* (29, 1396) = 1.322, *p* = 0.118].

Secondly, a three-way ANOVA was computed to determine the effects of cohort, gender, and age on composite scores for the MOT 4–6 sub-scales in order to gain more fine-grained insight into different aspects of motor development. Equality of covariance matrices was determined using Box’s test [*F* (756, 94,732.905) = 1.043, *p* = 0.202].

For the follow-up ANOVAs, homogeneity of variances was checked using Levene’s test (all *p*s > 0.074, except for Balance [*F* (29, 1396) = 1.594, *p* = 0.024], which was excluded from the analyses). Post hoc results were Bonferroni-corrected for multiple comparisons.

## 3. Results

A three-way ANOVA yielded no significant main effect of cohort [*F* (4, 1396) = 0.751, *p* = 0.557, *η_p_*^2^ = 0.002] for the overall MOT 4–6 T-scores (cf. Table 3), while the main effects of gender [*F* (1, 1396) = 14.491, *p* < 0.001, *η_p_*^2^ = 0.010; boys: *m* = 56.00, *sd* = 9.36; girls: *m* = 58.40, *sd* = 8.12] and age [*F* (2, 1396) = 3.569, *p* < 0.028, *η_p_*^2^ = 0.005; younger: *m* = 56.56, *sd* = 8.99; middle: *m* = 57.20, *sd* = 8.91; older: *m* = 58.74, *sd* = 7.88] were significant. None of the interaction effects were significant (two-way interactions: all *p*s > 0.076; three-way interaction: *p* = 0.292). Bonferroni corrected post hoc tests yielded a statistically significant difference between the groups of younger and older children (*p* = 0.003; both other *p*s > 0.067).

The three-way ANOVA revealed statistically significant multivariate effects of cohort [Pillai’s Trace = 0.062, *F* (28, 5572) = 3.155, *p* < 0.001, *η_p_*^2^ = 0.016], gender [Pillai’s Trace = 0.064, *F* (7, 1390) = 13.566, *p* < 0.001, *η_p_*^2^ = 0.064], and age [Pillai’s Trace = 0.067, *F* (14, 2782) = 6.886, *p* < 0.001, *η_p_*^2^ = 0.033] for the raw scores of the sub-scales of the MOT 4–6 (cf. Table 3). None of the interactions were significant (two-way interactions: all *p*s > 0.176; three-way interaction: *p* = 0.313).

The results of the ANOVAs and the follow-up tests for the sub-scales are presented in Table 4. Only the main effects are reported, as none of the two- or three-way interactions reached significance (all *p*s > 0.053).

## 4. Discussion

Given the relevance of motor activity for child development (Diamond, 2000, 2007; Stodden et al., 2008; Zimmer, 2013), and given that a number of studies found that during the COVID-19 pandemic, physical activity of children and adolescents was significantly reduced (Dunton et al., 2020; Haile et al., 2023; Moore et al., 2020; Pietrobelli et al., 2020; Xiang et al., 2020), the present study compared cohorts of preschoolers from the years 2015 to 2018, and 2020 to 2024 with respect to their fine and gross motor skills to assess the impact of the COVID-19 pandemic. Based on existing anonymized MOT 4–6 data (Zimmer, 2015) from a group of *n* = 1426 children enrolled in the daycare centers of the SportService des Landessportbund Brandenburg e.V., levels of motor skill development were analyzed in a cross-sectional study.

**Figure 1:**
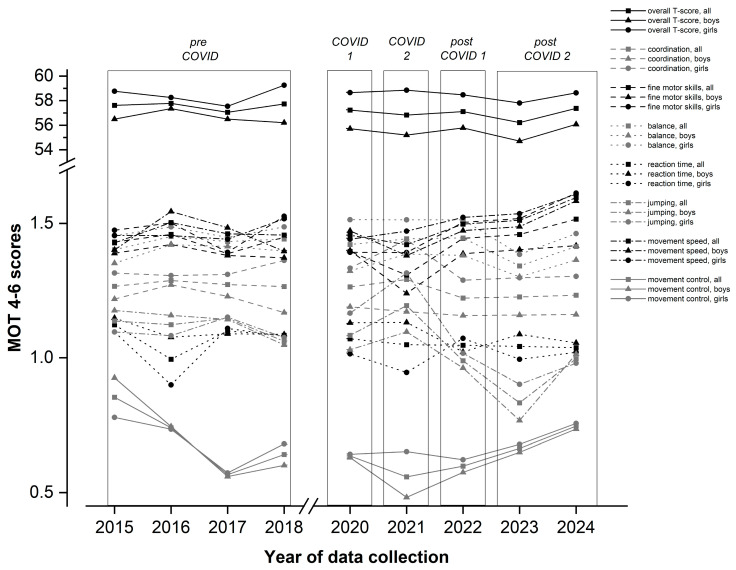
Children’s overall MOT 4–6 T-scores, as well as mean raw scores for the sub-scales by year (all children, and boys and girls, separately).

Overall, the results of the present study suggest that the restrictions imposed in the wake of the COVID-19 pandemic did not have a negative impact on the general motor performance level of five-year-olds in Germany. However, cohort effects can be observed with regard to individual motor performance dimensions (cf. Figure 1). A significant cohort effect was found for the motor dimension of jumping. Compared to the other cohorts, the jumping skills of the two post-COVID cohorts significantly declined, likely due to interruptions in institutionalized child daycare and general decreases in recreational sports activities during the pandemic. These restrictions occurred at a crucial phase of motor skill development, which explains the long-term nature of the effects. The somewhat counterintuitive finding of a brief improvement in jumping skills in the early phases of the pandemic is consistent with the results of the studies by Eberhardt et al. (2022) and Basterfield et al. (2022), who also reported short-term improvements in children’s jumping ability during the first lockdown phase. These effects can be explained with reference to studies that show temporary increases in children’s outdoor activity during the early phases of the COVID-19 pandemic (for an overview, see Liu et al., 2022; but also see Kourti et al., 2021). For example, Schmidt et al. (2022) showed that German children played outside for an average of 105 min per day during the lockdown in the spring of 2020, compared to only 75 min per day before the pandemic. However, this short-term increase in daily outdoor activity did not last. Rather, a decrease in physical activity was already observed in the second lockdown phase, resulting in an overall downward trend over the entire study period. So, even though children’s overall physical activity levels decreased during the pandemic, at least temporary increases in outdoor activity during the early lockdown phases may have contributed to the relative resilience of jumping skills. Additional opportunities for outdoor play, even outside formal institutional educational settings, may have promoted the development of those muscle groups that are relevant for the development of jumping ability (Pellegrini & Smith, 1998).

Regarding children’s fine motor skills, there is a significant cohort effect to the disadvantage of the COVID 2 cohort (cf. Figure 1). Similar findings are reported in the studies by Moore et al. (2020) and Lopes et al. (2020), which found pandemic-related declines in children’s fine motor skills and explained these findings with data suggesting that children were using more digital devices during lockdowns to the detriment of activities that promote the development of fine motor skills (such as arts and crafts or writing). The gender effects found for the domain of fine motor skills are consistent with the literature (Liong et al., 2015). Barnett et al. (2009) explain such findings with generally different inclinations of girls and boys with regard to physical activity. The authors point out that girls tend to pursue activities that promote fine motor skills, while boys are more inclined to pursue activities that promote the development of gross motor skills.

While no cohort effects were found for coordination, gender-specific effects were observed. Studies like the one by Lippi et al. (2020) support the assumption of Venetsanou and Kambas (2010), who suggest that certain motor skills are less susceptible to short-term changes in contextual conditions, such as those that occurred during the COVID-19 pandemic, due to their strong genetic determination. At the same time, Malina et al. (2004) explain gender-related differences with longer-term gender-specific preferences and tendencies in motor activity. Such differences are due to a combination of biological and socio-cultural factors. Girls often prefer activities that develop fine motor skills, while boys are more likely to participate in sports that require strength and speed, which, in turn, would explain the reversed gender effect for the sub-scale reaction time.

Finally, the significant age effects found across all sub-scales of the MOT 4–6 are self-explaining in that younger children generally performed worse than their respective older peers, independently from the motor domain under consideration (Zimmer, 2015).

In summary, the present results are well aligned with the findings of the few previous studies on the effects of the COVID-19 pandemic on motor development in preschool children, which also show rather complex patterns of affected and unaffected motor skills (Abe et al., 2022; Quenzer-Alfred, 2024; Rigó & Weyers, 2024). While no effects on general motor performance as measured by a standardized motor skills test were found for the groups of German 5-year-olds, a more detailed analysis revealed that the cohorts of children differed in their performance in certain motor domains. Such findings are relevant, on the one hand, for designing targeted support measures for children affected by the pandemic-related restrictions. On the other hand, the results indicate that even if no deficits can be found at the level of global motor performance, development in specific motor domains may well have suffered.

A number of limitations of the present study should be pointed out. First, the children involved in the present study were enrolled in daycare centers run by the Landessportbund Brandenburg e.V., which could indicate a certain self-selection bias. However, since these daycare centers are regular public childcare facilities that accept all children from the general population of the surrounding residential districts and do not have any special admission requirements, the present study should be representative of those areas of Germany that are similar in terms of the living environment of the children. Secondly, the MOT 4–6 primarily assesses motor skills and movement abilities but does not address fitness components such as physical endurance, muscle strength, and flexibility. Especially in light of the COVID-19 pandemic, where children had only reduced opportunities for physical activity due to school closures and limited access to recreational opportunities, a comprehensive assessment of children’s fitness levels would also be of great interest. Thirdly, one aspect that limits this study is the lack of test results from 2019. The data set available for secondary analysis only covers the years from 2015 to 2018 and from 2020 to 2024. Given that the missing year is considered a transition period between the pre-pandemic phase and the COVID-19 pandemic, it is not possible to clearly distinguish factors that were already in effect immediately before the pandemic from those that emerged in the wake of the pandemic. And, fourthly, for MOT 4–6, no gender-specific norms are available. However, looking at the results of the most recent MOT 4–6 standardization study (Zimmer, 2015), raw score differences in the sub-scales between girls and boys in favor of the girls are discernible, making a separation of test and pandemic-specific effects impossible. Further studies are needed to shed more light on the gender-specific effects of the COVID-19 pandemic on children’s motor development.

However, even taking these limitations into account, the present study provides insights into the effects of COVID-19-related restrictions on the development of motor skills in childhood. While no effects on overall motor performance as measured by the MOT 4–6, a standardized motor skills test, were found for the groups of preschoolers, a more detailed analysis revealed that the cohorts of children did differ with respect to two motor domains, i.e., jumping performance and fine motor skills. In view of the general lack of studies on this topic, the present study makes an important contribution to research on the impact of the COVID-19 pandemic on key developmental domains in the preschool years, which in turn is essential for the design of targeted remedial measures.

## Figures and Tables

**Table 1:** Characteristics of the sample.

Cohort	Year of Measurement	Sample			Age		
		Total *n*	Boys *n*	Girls *n*	Total *m* (*sd*)	Boys *m* (*sd*)	Girls *m* (*sd*)
Pre-COVID	2015	106	54	52	5.50 (0.30)	5.50 (0.30)	5.49 (0.31)
	2016	183	98	85	5.46 (0.29)	5.50 (0.29)	5.41 (0.30)
	2017	180	84	96	5.46 (0.29)	5.46 (0.28)	5.46 (0.29)
	2018	138	69	69	5.40 (0.27)	5.40 (0.28)	5.40 (0.26)
COVID 1	2020	198	96	102	5.49 (0.31)	5.47 (0.31)	5.51 (0.31)
COVID 2	2021	103	57	46	5.39 (0.28)	5.37 (0.28)	5.40 (0.29)
Post-COVID 1	2022	183	93	90	5.44 (0.28)	5.42 (0.27)	5.47 (0.30)
Post-COVID 2	2023	189	97	92	5.45 (0.26)	5.45 (0.24)	5.46 (0.28)
	2024	146	72	74	5.51 (0.18)	5.51 (0.28)	5.52 (0.28)
Overall							
		1426	720	706	5.46 (0.29)	5.45 (0.28)	5.46 (0.29)

Note: Age is expressed in decimal years.

**Table 2:** Sub-scales of the MOT 4–6 and exemplary tasks.

Sub-Scale	No. of Tasks	Examples of Diagnostic Tasks
Coordination	5	e.g., passing through a hoop without touching it or the ground
Fine motor skills	3	e.g., collecting matches with both hands simultaneously
Balance	5	e.g., balancing forward on a line (width: 10 cm)
Reaction time	2	e.g., catching a ring thrown in the air
Jumping	2	e.g., jumping over a rope (heights: 35 and 45 cm)
Movement speed	3	e.g., jumping sideways as often as possible within 10 s
Movement control	2	e.g., hitting a target on the wall with a small ball

Note: Most of the 17 diagnostics tasks load on more than one sub-scale of the MOT 4–6.

**Table 3:** Descriptive statistics.

	Pre-COVID		COVID 1		COVID 2		Post-COVID 1		Post-COVID 2	
	Boys *m* (*sd*)	Girls *m* (*sd*)	Boys *m* (*sd*)	Girls *m* (*sd*)	Boys *m* (*sd*)	Girls *m* (*sd*)	Boys *m* (*sd*)	Girls *m* (*sd*)	Boys *m* (*sd*)	Girls *m* (*sd*)
Mean overall T-scores	56.77 (8.46)	58.35 (7.98)	55.72 (8.80)	58.66 (7.93)	55.19 (10.15)	58.85 (8.49)	55.78 (9.86)	58.48 (8.61)	55.29 (10.25)	58.17 (8.23)
Mean scores for the sub-scales *										
Coordination	1.23 (0.39)	1.32 (0.38)	1.19 (0.37)	1.33 (0.37)	1.17 (0.48)	1.44 (0.38)	1.16 (0.43)	1.29 (0.42)	1.16 (0.40)	1.30 (0.38)
Fine motor skills	1.39 (0.37)	1.47 (0.38)	1.40 (0.33)	1.39 (0.33)	1.24 (0.43)	1.39 (0.41)	1.39 (0.38)	1.50 (0.36)	1.41 (0.39)	1.56 (0.34)
Balance	1.40 (0.34)	1.47 (0.30)	1.32 (0.34)	1.51 (0.31)	1.39 (0.42)	1.51 (0.39)	1.38 (0.40)	1.51 (0.31)	1.33 (0.35)	1.42 (0.33)
Reaction time	1.10 (0.50)	1.04 (0.50)	1.13 (0.45)	1.01 (0.49)	1.13 (0.49)	0.95 (0.57)	1.02 (0.52)	1.07 (0.51)	1.07 (0.53)	1.01 (0.49)
Jumping	1.13 (0.50)	1.10 (0.51)	0.97 (0.53)	1.19 (0.50)	1.10 (0.64)	1.32 (0.52)	0.96 (0.53)	1.02 (0.53)	0.87 (0.54)	0.94 (0.54)
Movement speed	1.47 (0.43)	1.46 (0.44)	1.47 (0.42)	1.44 (0.41)	1.38 (0.52)	1.47 (0.44)	1.47 (0.47)	1.52 (0.37)	1.53 (0.46)	1.57 (0.41)
Movement control	0.69 (0.58)	0.68 (0.51)	0.63 (0.57)	0.64 (0.52)	0.48 (0.50)	0.65 (0.48)	0.58 (0.57)	0.62 (0.46)	0.69 (0.57)	0.71 (0.53)

Note: * Composite raw scores divided by the number of sub-tests for each scale.

**Table 4:** Results of the follow-up ANOVAs.

	*df* 1, *df* 2	*F*	*p*	*η_p_* ^2^	Post Hoc Results ^‡^
Coordination					
Cohort	4, 1396	2.064	0.083	0.006	
Gender	1, 1396	24.258	<0.001	0.017	boys < girls
Age	2, 1396	15.860	<0.001	0.022	1 < 2 **; 2 < 3 *; 1 < 3 **
Fine motor skills					
Cohort	4, 1396	2.407	0.048	0.007	C < A/D *; C < E **
Gender	1, 1396	15.015	<0.001	0.011	boys < girls
Age	2, 1396	25.268	<0.001	0.035	1 < 2 **; 1 < 3 **
Reaction time					
Cohort	4, 1396	0.608	0.657	0.002	
Gender	1, 1396	4.419	0.036	0.003	boys > girls
Age	2, 1396	15.369	<0.001	0.022	1 < 2 **; 2 < 3 *; 1 < 3 **
Jumping					
Cohort	4, 1396	11.679	<0.001	0.032	D < A/C *; E < A/B/C **
Gender	1, 1396	2.385	0.123	0.002	
Age	2, 1396	14.040	<0.001	0.020	1 < 2 **; 2 < 3 *; 1 < 3 **
Movement speed					
Cohort	4, 1396	1.266	0.281	0.004	
Gender	1, 1396	0.316	0.574	0.000	
Age	2, 1396	28.710	<0.001	0.040	1 < 2 **; 1 < 3 **
Movement control					
Cohort	4, 1396	1.619	0.167	0.005	
Gender	1, 1396	0.317	0.573	0.000	
Age	2, 1396	25.232	<0.001	0.035	1 < 2 **; 2 < 3 *; 1 < 3 **

Note: * *p*s ≤ 0.050, ** *p*s ≤ 0.001. ^‡^ Only significant effects after Bonferroni correction are reported. For age groups, 1 = younger, 2 = middle, and 3 = older. For cohorts, A = pre-COVID, B = COVID 1, C = COVID 2, D = post-COVID 1, and E = post-COVID 2.

## Data Availability

The data set is not available to the public due to privacy restrictions. While parents of the children involved in this study gave their consent for primary and secondary analyses of the data and publication of aggregated results, no consent was given for publication of the original data.

## Abbreviations

The following abbreviations are used in this manuscript:

COVID-19	Coronavirus disease 2019
MOT 4–6	Motoriktest für vier- bis sechsjährige Kinder
